# Development of Rapid Alkaline Lysis–Polymerase Chain Reaction Technique for Authentication of Mithun (*Bos frontalis*) and Yak (*Bos grunniens*) Species

**DOI:** 10.3390/molecules30040934

**Published:** 2025-02-18

**Authors:** Moon Moon Mech, Hanumant Singh Rathore, Jyoti Jawla, Nagappa Karabasanavar, Sapunii Stephen Hanah, Harshit Kumar, Vikram Ramesh, Arockiasamy Arun Prince Milton, Vijay Kumar Vidyarthi, Mihir Sarkar, Girish Patil Shivanagowda

**Affiliations:** 1 School of Engineering and Technology, Nagaland University, Kohima 797004, Nagaland, India; moonmoonmech11@gmail.com (M.M.M.); hanu7862007@yahoo.com (H.S.R.); vidyarthi64@gmail.com (V.K.V.); 2 ICAR-Indian Veterinary Research Institute, Izatnagar 243122, Uttar Pradesh, India; jawlajyoti@gmail.com; 3 Department of Veterinary Public Health and Epidemiology, Veterinary College, KVAFSU, Vidyanagar, Hassan 573202, Karnataka, India; pub.nag@kvafsu.edu.in; 4 ICAR-National Research Centre on Mithun, Medziphema 797106, Nagaland, India; shanah@rediffmail.com (S.S.H.); harshit.kumar@icar.gov.in (H.K.); vikki955@gmail.com (V.R.); 5 Division of Animal and Fisheries Sciences, ICAR Research Complex for NEH Region, Umiam 793103, Meghalaya, India; vetmilton@gmail.com; 6 ICAR-National Research Centre on Yak, Dirang 790101, Arunachal Pradesh, India; director.nrcy@icar.gov.in

**Keywords:** Mithun, yak, meat, speciation, alkaline lysis, PCR

## Abstract

*Bos frontalis* (Mithun) and *Bos grunniens* (yak) are crucial to the culture, food security, and economy of Southeast Asia, especially in India and China, respectively. Their genetic closeness to *Bos indicus* (indicine cattle) and *Bos taurus* (taurine cattle) necessitates precise methods for meat origin authentication. This study introduces a DNA-based technique to distinguish Mithun and yak species using the alkaline lysis (AL) protocol for DNA extraction, followed by species-specific polymerase chain reaction (PCR) to amplify unique mitochondrial D-loop regions, yielding 489 bp and 422 bp amplicons, respectively. The AL-PCR method showed high specificity for both species, with no cross-amplification with other related species. The method’s effectiveness was validated across various sample preparations, including raw, cooked, autoclaved, microwaved, and fried samples. The AL-PCR assay is highly sensitive, detecting as little as 1 pg of Mithun DNA and 100 pg of yak DNA, and can identify down to 0.1% of these species in binary mixtures. This approach is rapid and cost-effective, offering significant benefits for consumer protection, promoting Mithun and yak farming, and addressing food safety and traceability issues.

## 1. Introduction

The Mithun is primarily a meat animal, while the yak is used for both meat and milk production. Both are hill bovines and are highly preferred for consumption by the tribal populations of the region. However, the availability of Mithun and yak meat is limited due to their small populations, unorganized production, and processing practices. As a result, there are instances of misrepresentation of Mithun and yak meat with other, lower-cost meats. Meat authentication is a crucial aspect of food safety and quality, especially for religious and cultural practices that require the use of specific animal species [1]. The identification of meat species is essential to prevent consumer fraud and ensure the authenticity of the products. The NEH region of India, a global biodiversity hotspot, is rich in both plant diversity and animal genetic resources [2]. Among its unique bovine species, Mithun and yak are noteworthy for their economic value and cultural significance. Mithun thrives in the subtropical rainforests, while yaks are adapted to high-altitude, cold environments. These species not only represent biological interests but also hold profound cultural and economic importance for the indigenous communities of the NEH region and parts of Southeast Asia. For tribal communities, owning a Mithun is a source of immense pride. It signifies not just wealth, but also an individual’s social standing within the community [3]. Live Mithuns are traditionally offered as bride price in most tribes of Arunachal Pradesh and Nagaland [4]. Particularly in the traditional practices of the Monpa tribe, virtually all anatomical components of the yak hold cultural and religious significance. Traditionally, indigenous communities incorporate the meat of Mithun and yak into matrimonial ceremonies, communal feasts, and other significant social gatherings [2].

Meat from Mithun and yak is nutritionally rich, featuring low fat, high protein, and a unique amino acid profile, making it highly valued for both its health benefits and distinctive flavor [5,6]. This nutritional appeal, combined with its cultural significance, underscores the necessity for precise species authentication to prevent adulteration and ensure consumers receive genuine products. Local populations residing in the NEH region of India adhere to customary dietary practices that prioritize meat as a staple component [7]. The rich cultural and traditional diversity amongst NEH communities has fostered the development of a vast array of indigenous meat products. Furthermore, meat consumption patterns and expenditure within this region are demonstrably 2–3 times higher compared to the national average [8], highlighting the significant role that meat and meat products play in the region’s dietary landscape. The meat of both animals is recognized for its marbled texture and delectable type of beef, which local people have prized above that of cattle.

The morphological similarities and hybridization potential of Mithun and yak present a challenge for accurate identification. Current methods are limited and do not adequately address the need for reliable authentication in this region. Previous studies have successfully employed PCR techniques to detect the presence of porcine DNA in meat products [9] and to verify the authenticity of meat from game animal species [1]. The present study builds upon this existing knowledge to establish a PCR-based protocol for the identification of Mithun and Yak meat.

Further, extensive research has explored various facets of Mithun biology, encompassing genetics, nutrition, anatomy, and physiology [10]. However, dedicated efforts to develop species-specific identification methods for Mithun meat are currently scarce. Only one study, based on mitochondrial 12S rRNA analysis, has been conducted [11]. Conversely, a limited number of analytical techniques utilizing molecular biological principles have been established for yak meat identification worldwide [12,13]. A critical gap exists in the research on bovine meat adulteration. Historically, investigations have largely neglected Mithun entirely and, to a lesser extent, yak. Notably, there is an absence of studies that examine adulteration involving both Mithun and yak simultaneously. This lack of comprehensive research creates a vulnerability, potentially allowing the adulteration of these species’ meat with close hybrids to go undetected.

Over the past two decades, a multitude of analytical methods have been developed for meat speciation, which include anatomical, histological, microscopic, organoleptic, chemical, electrophoretic, chromatographic, and immunological techniques. Nonetheless, inherent limitations in these approaches have shifted the adoption of DNA-based molecular techniques over the other methods, owing to the versatile and discriminatory nature of DNA, especially the mitochondrial targets [14].

Various assays for species identification in food predominantly rely on PCR-based DNA analysis techniques such as polymerase chain reaction–restriction fragment length polymorphism (PCR-RFLP) [15], real-time PCR [16], and DNA mini-barcoding coupled with next-generation sequencing (NGS) [17], which have proven effective in detecting minute DNA quantities and are known for their high precision. However, these methodologies are encumbered by intricate procedural requirements, the necessity for costly instrumentation, and protracted processing durations, thus constraining their applicability in resource-limited laboratory settings.

Species-specific PCR represents a valuable genetic method for the novel identification of various meat species. This technique offers several advantages over other PCR-based assays for meat product analysis [18]. Species-specific PCR demonstrates high precision and sensitivity in detecting DNA from a broad range of mammalian and avian species, even in raw and thermally treated meats [19]. Additionally, species-specific PCR is a relatively inexpensive and time-efficient method, making it a favorable choice for routine meat product authentication.

This method relies on the principle of primer hybridization and subsequent in vitro amplification of target DNA sequences. Specific oligonucleotide primers flank the target region, enabling the enzymatic synthesis of millions of copies of the desired fragment. Following amplification, agarose gel electrophoresis is employed to verify the size of the amplified fragment, thus confirming successful target DNA amplification. Furthermore, this method eliminates the need for downstream processes such as additional sequencing or restriction enzyme digestion of the PCR amplicons [20]. This simplifies the workflow and reduces analysis time.

Mitochondrial DNA (mtDNA) fingerprinting of Indigenous bovines in North-East India remains a relatively understudied area, particularly for Mithun and yak. Reports on the development of molecular techniques for species identification of Mithun and yak meat are scarce. The current investigation introduces a novel AL-PCR method combining a rapid DNA extraction with species-specific primers targeting the mitochondrial D-loop region. This study aims to develop a PCR-based protocol to accurately differentiate between Mithun and yak meat, thereby addressing a critical gap in existing meat authentication methods. This is the first approach aimed at the simultaneous identification of Mithun and yak meat, promising a practical and efficient solution for authenticating the culturally and nutritionally significant species.

## 2. Results

### 2.1. Comparative Analysis of DNA Isolation Methods for PCR

Two DNA extraction protocols, alkaline lysis (AL) and the Wizard^®^ Genomic DNA Purification Kit were evaluated for Mithun and yak meat samples. From the AL method, for raw meat samples (*n* = 6), the DNA yield for Mithun ranged from 22.2 to 123.8 ng/µL, with an OD_260_/OD_280_ ratio of 1.29–1.63. Similarly, yak DNA yield ranged from 28.9 to 161.2 ng/µL, with an OD_260_/OD_280_ ratio of 1.38–1.69. While the kit method with a 20 mg sample, requiring 2 hrs, yielded 147.9 to 437.2 ng/µL with an OD_260_/OD_280_ ratio of 1.7–1.9 for raw Mithun meat samples and raw yak meat samples, the DNA yield ranged from 137.1 to 317.5 ng/µL with an OD_260_/OD_280_ ratio of 1.68–1.85.

Across various heat treatments (60 °C, 80 °C, 100 °C, 121 °C), microwave cooking, and deep frying, from the AL method, Mithun DNA yield ranged from 33.5 to 80.1 ng/µL, with an OD_260_/OD_280_ ratio of 1.42–2.43, while yak DNA yield ranged from 25.7 to 44.1 ng/µL, with an OD_260_/OD_280_ ratio of 1.3–2.28. From the kit method, Mithun DNA yield ranged from 23.7 to 287 ng/µL with an OD_260_/OD_280_ ratio of 1.9–2.36, while yak DNA yield ranged from 81.7 to 179.8 ng/µL with an OD_260_/OD_280_ ratio of 1.9–2.25.

### 2.2. Optimization of Species-Specific PCR of Mithun and Yak

**Figure 1:**
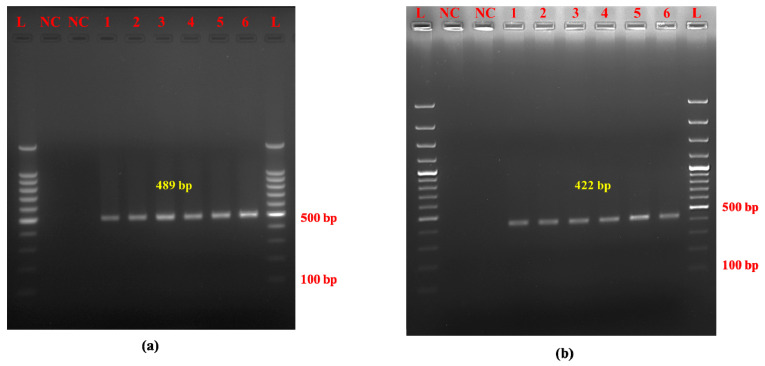
Species-specific amplification of mitochondrial D-loop region of (**a**) Mithun and (**b**) yak using novel primers. Note. (**a**) L: 100 bp DNA ladder, NC: negative control, 1–6: raw Mithun meat samples (*n* = 6). (**b**) L: 100 bp DNA ladder, NC: negative control, 1–6: raw yak meat samples (*n* = 6).

Optimization resulted in a single PCR product specific for Mithun with a size of 489 bp and a distinct yak-specific amplicon of 422 bp. The optimal annealing temperatures for amplifying Mithun and yak DNA were determined to be 52 °C and 50 °C, respectively (Figure 1a,b).

### 2.3. Sensitivity and Specificity of Mithun and Yak Species-Specific PCR

**Figure 2:**
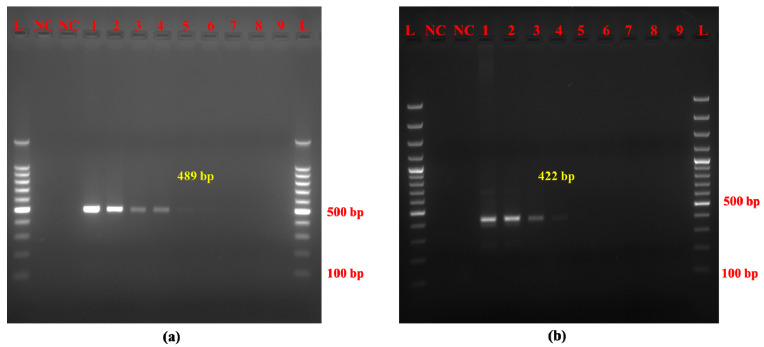
Sensitivity of (**a**) Mithun and (**b**) yak AL-PCR. Note. (**a**,**b**) L: 100 bp DNA ladder, NC: negative control, 1: 100 ng DNA, 2: 10 ng DNA, 3: 1 ng DNA, 4: 100 pg DNA, 5: 10 pg DNA, 6: 1 pg DNA, 7: 100 fg DNA, 8: 10 fg DNA, 9: 1 fg DNA.

**Figure 3:**
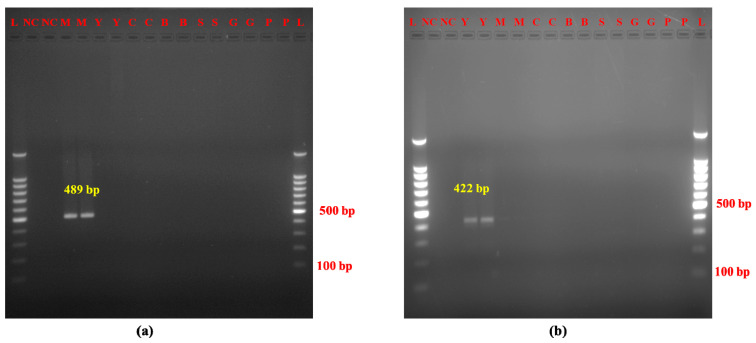
Specificity of (**a**) Mithun and (**b**) yak AL-PCR. Note. (**a**) L: 100 bp DNA ladder, NC: negative control, M: Mithun, Y: yak, C: cattle, B: buffalo, S: sheep, G: goat, P: pig. (**b**) L: 100 bp DNA ladder, NC: negative control, Y: yak, M: Mithun, C: cattle, B: buffalo, S: sheep, G: goat, P: pig.

Our optimized species-specific AL-PCR assay targeting Mithun and yak exhibited high sensitivity and specificity. The limit of detection (LOD) achieved was 1 pg for Mithun DNA and 100 pg for yak DNA (Figure 2a,b). The newly developed Mithun and yak species-specific PCR assay displayed high specificity, as shown by the complete absence of cross-amplification with closely related species (Figure 3a,b). This specificity is facilitated by the inherent variability within mitochondrial DNA sequences, allowing for the design of species-specific targets as exemplified by the current AL-PCR assay.

### 2.4. Suitability of Mithun and Yak AL-PCR for Admixed Meat

**Figure 4:**
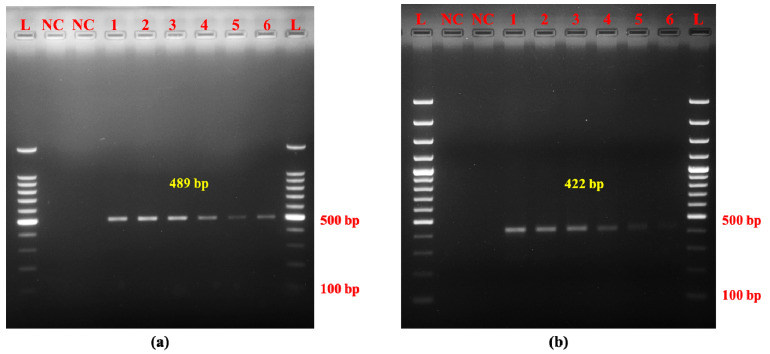
Evaluation of the suitability of (**a**) Mithun and (**b**) yak AL-PCR for admixed meat samples. Note. (**a**) L: 100 bp DNA ladder, NC: negative control, 1: 20%, 2: 10%, 3: 5%, 4: 1%, 5: 0.5%, 6: 0.1% (Mithun DNA at different concentrations). (**b**) L: 100 bp DNA ladder, NC: negative control, 1: 20%, 2: 10%, 3: 5%, 4: 1%, 5: 0.5%, 6: 0.1% (yak DNA at different concentrations).

The binary meat blend effectively amplified Mithun and yak DNA down to a concentration as low as 0.1% (Figure 4a,b), underscoring the applicability of the newly developed AL-PCR assay for detecting adulteration when Mithun and yak meat are combined with meats from other species, even at minimal levels.

### 2.5. Suitability of Mithun and Yak AL-PCR for Cooked Meat Samples

**Figure 5:**
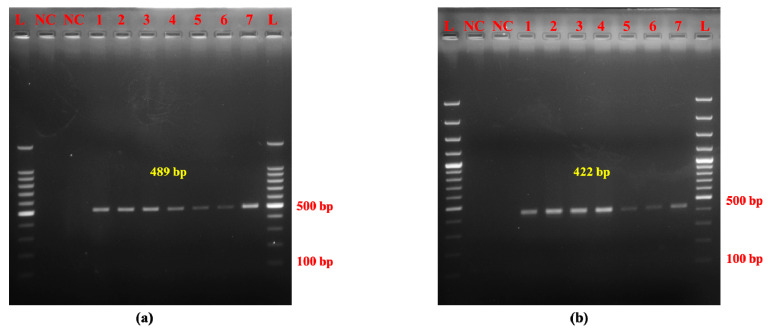
Evaluation of the suitability of (**a**) Mithun and (**b**) yak AL-PCR for cooked meat samples. Note. (**a**) L: 100 bp DNA marker, NC: negative control, 1: raw Mithun meat, 2: Mithun meat cooked at 60 °C for 30 min, 3: Mithun meat cooked at 80 °C for 30 min, 4: Mithun meat cooked at 100 °C for 30 min, 5: Mithun meat cooked in the microwave oven at 300 watts for 30 min, 6: Mithun meat processed in an autoclave at 121 °C and 15 psi for 30 min, 7: Mithun meat fried in vegetable oil at 177–180 °C for 5–8 min. (**b**) L: 100 bp DNA marker, NC: negative control, 1: raw yak meat, 2: yak meat cooked at 60 °C for 30 min, 3: yak meat cooked at 80 °C for 30 min, 4: yak meat cooked at 100 °C for 30 min, 5: yak meat cooked in microwave oven at 300 watts for 30 min, 6: yak meat processed in autoclave at 121 °C and 15 psi for 30 min, 7: yak meat fried in vegetable oil at 177–180 °C for 5–8 min.

The efficacy of the AL-PCR assay for Mithun and yak was evaluated using cooked meat samples. This experiment validated the assay’s ability to detect Mithun and yak DNA not only in raw meat but also in thermally processed samples up to 121 °C, as evidenced by the amplification of 489 bp and 422 bp amplicons specific for Mithun and yak, respectively (Figure 5a,b).

## 3. Discussion

This study presents a novel application of species-specific PCR for the accurate identification of Mithun (*Bos frontalis*) and yak (*Bos grunniens*) meat. This method addresses a significant gap in the current capabilities for meat authentication, particularly in the context of the unique bovine species of Southeast Asia (esp. India and China). The successful implementation of species-specific PCR in this study demonstrates its utility in ensuring the authenticity of meat products, which is crucial for both consumer protection and cultural practices.

### 3.1. Efficiency and Practicality of the AL-PCR Method

Two methods of DNA extraction were compared and evaluated. Although the DNA extracted from the AL method had low DNA purity and yield as compared to the kit method, the results obtained from this method could isolate sufficient DNA for downstream applications even after heat processing. Importantly, the DNA templates obtained using the AL method were suitable for subsequent PCR amplification. Despite some drawbacks, alkaline lysis is still widely used for many routine DNA extraction protocols due to its simplicity and effectiveness for certain types of samples.

The mitochondrial D-loop region was chosen as the target gene because of its hypervariability, primarily caused by indels and tandemly repeated sequences, which allows for effective species discrimination. Additionally, this region evolves more rapidly than other mitochondrial protein-coding regions due to weaker selective constraints, making it suitable for designing specific primers for species identification [21,22]. The presence of multiple circular mitochondrial DNA copies per cell also enhances PCR amplification, even in degraded or processed samples. Both Mithun and yak species-specific PCR assays utilizing newly designed primers successfully generated a single, diagnostic signal, confirming the functionality of the AL-DNA extraction and PCR combination for species identification.

The AL method offers several advantages over traditional or commercially available DNA extraction kits. It is remarkably straightforward, expeditious, and cost-effective, making it particularly suitable for laboratories with limited resources. Since 1998, researchers have pioneered the AL technique for rapid DNA extraction from whole blood [23]. Subsequently, this method underwent further adaptations for extracting DNA from meat [24], achieving completion within 30 min at 75 °C. The adaptability of the AL-based DNA extraction method is further emphasized by its successful application in extracting DNA from various meat sources [25,26,27].

The entire procedure can be completed in a single tube with minimal reagents (alkali and buffer), utilizing a standard water or dry bath. This single-tube approach minimizes contamination risks and eliminates the need for expensive equipment. Moreover, this method holds the potential to significantly increase sample throughputs in laboratories handling large volumes of samples, thereby reducing sample turnaround time (TAT). The versatility and robustness of the AL method extend beyond raw samples, as it isolates DNA from thermally processed samples (cooked up to 121 °C), emphasizing its broad applicability for meat authentication purposes.

### 3.2. Specificity and Sensitivity of the Species-Specific PCR Assay

A novel species-specific PCR assay was developed and optimized for the identification of Mithun and yak. This assay utilizes newly designed primers targeting the conserved mitochondrial D-loop region of both species. Several studies have established the efficacy of shorter amplicons in enhancing sample detection probability [28]. Mitochondrial 12S rRNA gene sequence analysis has been established as a reliable method for differentiating Mithun from other commonly consumed red meats such as cattle, buffalo, sheep, and goats [11]. Another study reported a multiplex PCR assay for the rapid detection of yak and cattle meat, employing a combination of three primers targeting the mitochondrial 12S rRNA gene [6].

Remarkably, this study represents the first report on the limit of detection (LOD) for Mithun in meat detection systems. Conversely, yak LOD has been previously explored, with varying results. Variable mitochondrial DNA regions were targeted in eight animals, including yak, which achieved a 491 bp amplicon with an analytical sensitivity ranging from 5 to 20 pg [29]. In another study, yak DNA sensitivity of 0.04 ng/µL was recorded by employing SNP markers to distinguish meat from yak and cattle by application of a multiplex allele-specific PCR (AS PCR) and capillary electrophoresis (CE) method [13].

For DNA-based animal species identification techniques, LOD exhibits significant variability. This is attributed to a complex interplay of factors including the target species, the chosen DNA target sequence, and the inherent characteristics of the sample itself [30]. Assay design also plays a crucial role in influencing the ultimate LOD achieved.

The newly developed Mithun and yak species-specific PCR assay displayed high specificity, as shown by the complete absence of cross-amplification with closely related species (Figure 3a,b). This specificity is facilitated by the inherent variability within mitochondrial DNA sequences, allowing for the design of species-specific targets as exemplified by the current AL-PCR assay. Significantly, the primer design strategy utilized the intra-species sequence conservation and inter-species sequence divergence of mitochondrial DNA. This intrinsic robustness of mitochondrial DNA targets translates to their continued discernibility in processed food products, even persisting after thermal treatments like heat processing. These unique genetic sequences offer optimal suitability for species identification [31].

### 3.3. Validation with Admixed and Cooked Meat Samples

The efficacy of the AL-PCR assay for Mithun and yak was evaluated using cooked meat samples. The ability of the AL-PCR assay to detect Mithun and yak DNA in binary mixtures down to a concentration of 0.1% underscores its potential for use in adulteration detection. This is particularly relevant in the context of the NEH region where traditional meat products may be subjected to substitution or mixing with other species, either inadvertently or fraudulently. The successful amplification of species-specific DNA in both raw and cooked samples further confirms the assay’s applicability in real-world scenarios, including those involving complex meat matrices and various cooking methods. A real-time PCR method was established for the detection of yak components in meat products [32], where the assay, being specific and highly sensitive, has a high potential for rapid detection of yak components present in raw or processed meat. However, instances of target DNA degradation and reduction in PCR signals were observed in samples processed beyond 121 °C, as reported in previous studies [33]. The mitochondrial D-loop region, a commonly employed mitochondrial target, exhibited successful amplification even at elevated temperatures, and this reinforces the D-loop region of mtDNA as a robust target for meat species identification [24].

## 4. Materials and Methods

### 4.1. Collection of Meat Samples

Mithun meat samples (*n =* 6) were collected from the municipal slaughterhouse located in Dimapur, Nagaland, India. Tissue samples of yak (*n =* 6) and sheep (*n =* 6) were collected from the post-mortem hall of ICAR-National Research Centre on Yak (Dirang, India) and the College of Veterinary Sciences and Animal Husbandry (Jalukie, India). Authentic cattle, buffalo, goat, and pig meat samples (*n =* 6 each) were procured from local meat shops in Dimapur, Nagaland. All the meat samples (~300 g) were individually collected and transported under refrigerated conditions to the laboratory at ICAR-National Research Centre on Mithun (Medziphema, Nagaland, India). Upon arrival, samples were either immediately processed for DNA extraction using the AL method or stored at −20 °C for further analysis.

### 4.2. DNA Extraction

Total nucleic acid was extracted from all samples using the alkaline lysis method [21]. Briefly, 500 mg of fresh meat tissue was homogenized in an autoclaved mortar and pestle with eight volumes (4 mL) of 0.2 N sodium hydroxide solution. Then, 10 µL of the resulting lysate was then mixed with 40 µL of 0.2 N NaOH and incubated at 75 °C for 20 min in a dry bath incubator (ACCUBLOCK™, Digital dry bath, Labnet International, Inc., Edison, NJ, USA). Following thermal lysis, the reaction was neutralized by adding 360 µL of 0.04 M Tris-HCl (pH 7.75). Additionally, DNA was isolated from tissue samples using the commercially available Wizard^®^ Genomic DNA Purification Kit (Promega, Madison, WI, USA) according to the manufacturer’s instructions. The extracted nucleic acids were stored immediately at −20 °C until further analysis. The concentration and purity of the isolated nucleic acids were assessed using a spectrophotometer (NanoDrop2000, ThermoFisher Scientific, Waltham, MA, USA) by measuring the OD_260/280_ nm ratio.

### 4.3. Designing of New Primers for Mithun and Yak

Mitochondrial D-loop sequences of *Bos frontalis* (Mithun) and *Bos grunniens* (yak) were retrieved from GenBank^®^ (National Institutes of Health, https://www.ncbi.nlm.nih.gov/genbank, accessed on 17 May 2023). Sequence alignment was performed using the Mega Align program (Lasergene Version 17.4, DNAstar, Inc., Madison, WI, USA). Regions exhibiting interspecific nucleotide variation and intraspecific homology were identified and targeted for primer design. Mithun- and yak-specific primers were designed using the ‘Primer-Select’ program (Lasergene software version 17.4, DNAstar, Inc.) and evaluated for species specificity and potential cross-reactivity through in silico analysis using the local nucleotide alignment tool ‘BLAST’ (http://www.ncbi.nlm.nih.gov/blast, accessed on 17 May 2023). The probable amplicon size of the Mithun-specific primer set was 489 bp, whereas the probable amplicon size of yak specific primer set was 422 bp. These primers were custom synthesized (GCC Biotech India Pvt. Ltd., Kolkata, West Bengal, India) and employed for PCR amplification. The complete primer sequences for both species are provided in Table 1 and Supplementary Figures S1 and S2.

### 4.4. Standardization of Mithun and Yak PCR

PCR amplification for both Mithun and yak was performed in a 25 µL reaction mixture containing 12.5 µL of 2X PCR Master Mix (Promega, Madison, WI, USA), 10 µM each of forward and reverse primers (1 µL each) synthesized by GCC Biotech (Kolkata, West Bengal, India), 50 ng of DNA template, and nuclease-free water (Promega, Madison, WI, USA) to achieve the final volume. Following brief centrifugation in a microcentrifuge, the reaction tubes were placed in a veriti 96-well thermal cycler (Applied Biosystems, Waltham, MA, USA) programmed with the following cycling conditions: initial denaturation at 95 °C for 5 min, followed by 30 cycles of denaturation at 95 °C for 30 s, annealing at different gradient temperatures (44–54 °C for Mithun, 46–56 °C for yak) for 30 s, and extension at 72 °C for 30 s. A final extension step at 72 °C for 5 min was included, and the PCR products were held at 4 °C until subsequent electrophoresis. Analysis of the PCR products was performed on 2% agarose gels (SERVA, Heidelberg, Germany) prepared with 1X TAE buffer (Promega, USA). The gels were stained with 0.5 µg/mL ethidium bromide (HiMedia, Thane, Maharashtra, India) and electrophoresed at 75 V alongside a 100 bp DNA ladder (Promega, Madison, Wisconsin, USA, and GCC Biotech, Kolkata, West Bengal, India) for visualization of amplicon sizes. Images of the gels were captured using a G: BOX F3 Gel Documentation System (Syngene, Cambridge, UK).

### 4.5. Sensitivity and Specificity of Mithun and Yak

The analytical sensitivity of the AL-PCR assay for Mithun and yak was evaluated using ten-fold serial dilutions of the target DNA, ranging from 100 ng/µL to 1 fg/µL. The LOD was determined as the lowest dilution that resulted in a visible amplicon after 30 cycles of PCR in a 25 µL reaction mixture, following optimization. To evaluate the specificity of the assays, DNA templates from relevant animal species were employed. For the Mithun assay, this included yak, cattle, buffalo, sheep, goats, and pigs. Similarly, the yak assay was tested against DNA from Mithun, cattle, buffalo, sheep, goats, and pigs to ensure the absence of cross-amplifications.

### 4.6. AL-PCR of Mithun and Yak for Admixed and Cooked Meat Samples

To assess the discriminatory power of the AL-PCR assay in complex mixtures, binary admixtures of Mithun and yak meat were prepared with cattle meat at varying ratios: 20:80, 10:90, 5:95, 1:99, 0.5:99.5, and 0.1:99.9 (Mithun/yak to cattle). DNA was extracted from each binary sample and subjected to species-specific PCR for both animals.

Furthermore, the impact of heat processing on assay performance was evaluated. Fresh meat samples from both Mithun and yak were subjected to various thermal treatments mimicking diverse culinary practices: simmering (dry heat at 60 °C for 30 min), boiling (dry heat at 80 °C and 100 °C for 30 min), pressure-steaming (121 °C and 15 psi for 30 min), microwave cooking (300 W for 30 min), and deep frying (vegetable oil at 177–180 °C for 5–8 min). Following processing, DNA from these samples was isolated and subjected to a species-specific PCR assay to determine the ability to detect Mithun and yak genetic material under various cooking conditions.

## 5. Conclusions

This study presents a novel and user-friendly AL-PCR assay for the rapid identification of Mithun and yak species. The assay targets the mitochondrial D-loop region using newly designed primers and offers several advantages for routine sample analysis. Importantly, the AL-PCR assay applies to raw, cooked (up to 121 °C), and adulterated meat samples. It demonstrates high sensitivity, detecting Mithun and yak meat adulteration in other meat species down to 0.1% levels. Additionally, the assay exhibits a detection limit of 1 pg for Mithun DNA and 100 pg for yak DNA. This study establishes a groundwork for further exploration of these iconic species and offers a valuable tool for quality control in food laboratories and regulatory bodies. The implementation of AL-PCR has the potential to promote transparency, accountability, and trust within the meat supply chain.

## Figures and Tables

**Table 1:** Details of primers used in this study.

Species	Target Region	Sequences (5′-3′)	Product Length
Mithun (*Bos frontalis*)	Mitochondrial D-loop	Forward-CCT CCC GCA CCA CTA CAG AAT A	489 bp
		Reverse-CCA AGC ATC CCC CAA AAT AAA AA	
Yak (*Bos grunniens*)	Mitochondrial D-loop	Forward-TAA ATG TAA AGA GCC TCA CCA GTA	422 bp
		Reverse-ATT AAA TAG CGA CCC CCA CAG TTC	

## Data Availability

Data will be made available on request.

## Supplementary Materials

The following supporting information can be downloaded at https://www.mdpi.com/article/10.3390/molecules30040934/s1. Figure S1: Primer sequences for Mithun (*Bos frontalis*). Figure S2: Primer sequences for Yak (*Bos grunniens*).
