# Supplementary material for: Development of Rapid Alkaline Lysis–Polymerase Chain Reaction Technique for Authentication of Mithun (Bos frontalis) and Yak (Bos grunniens) Species
Source: Molecules. 2025 Feb 18;30(4):934. doi: 10.3390/molecules30040934 (PMC11857932; doi:10.3390/molecules30040934)
Supplement: Supplementary file 1 [file molecules-30-00934-s001.zip › molecules-3339188-supplementary.pdf]

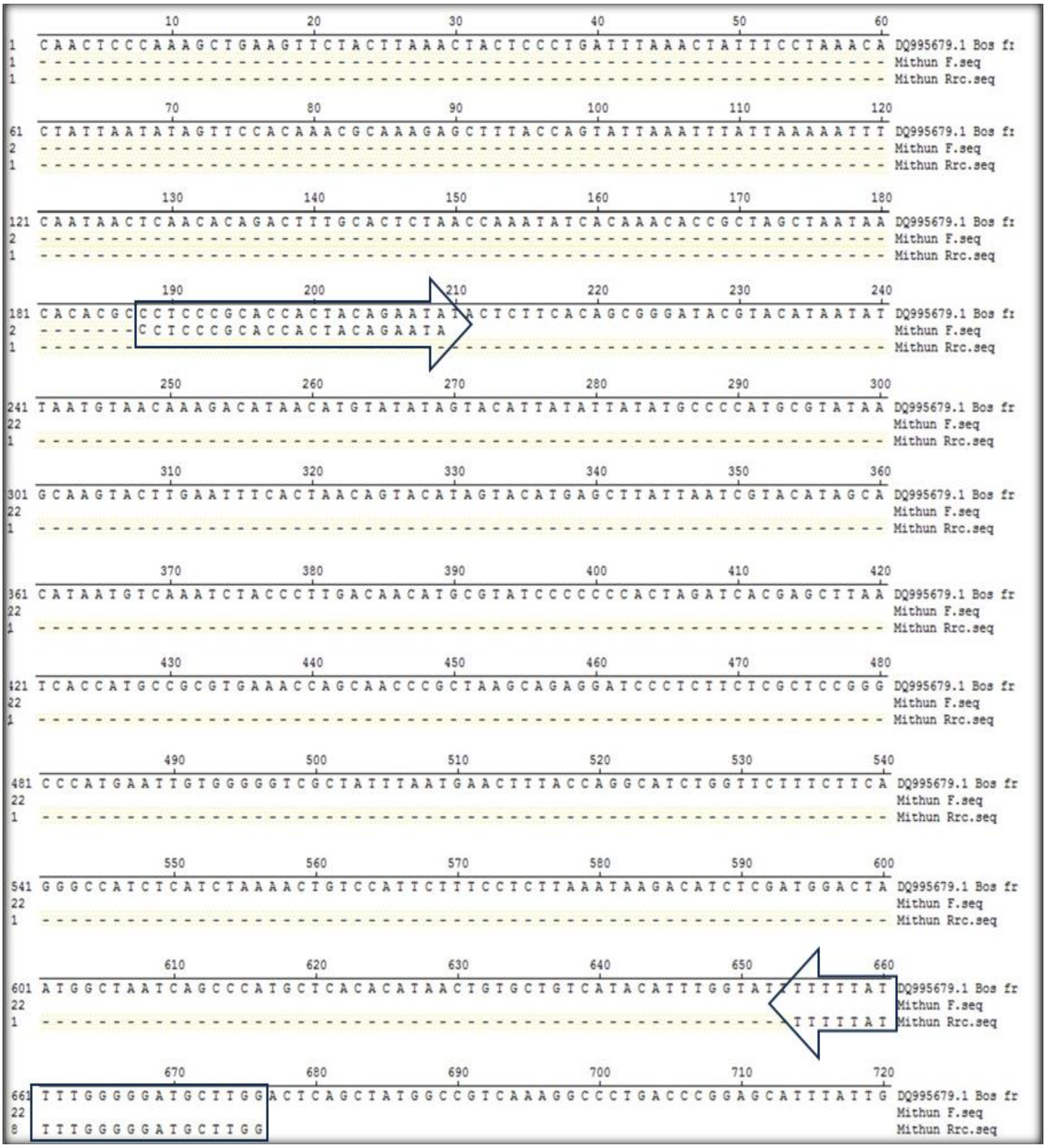

Figure S1. The primer sequences for Mithun (*Bos frontalis*)

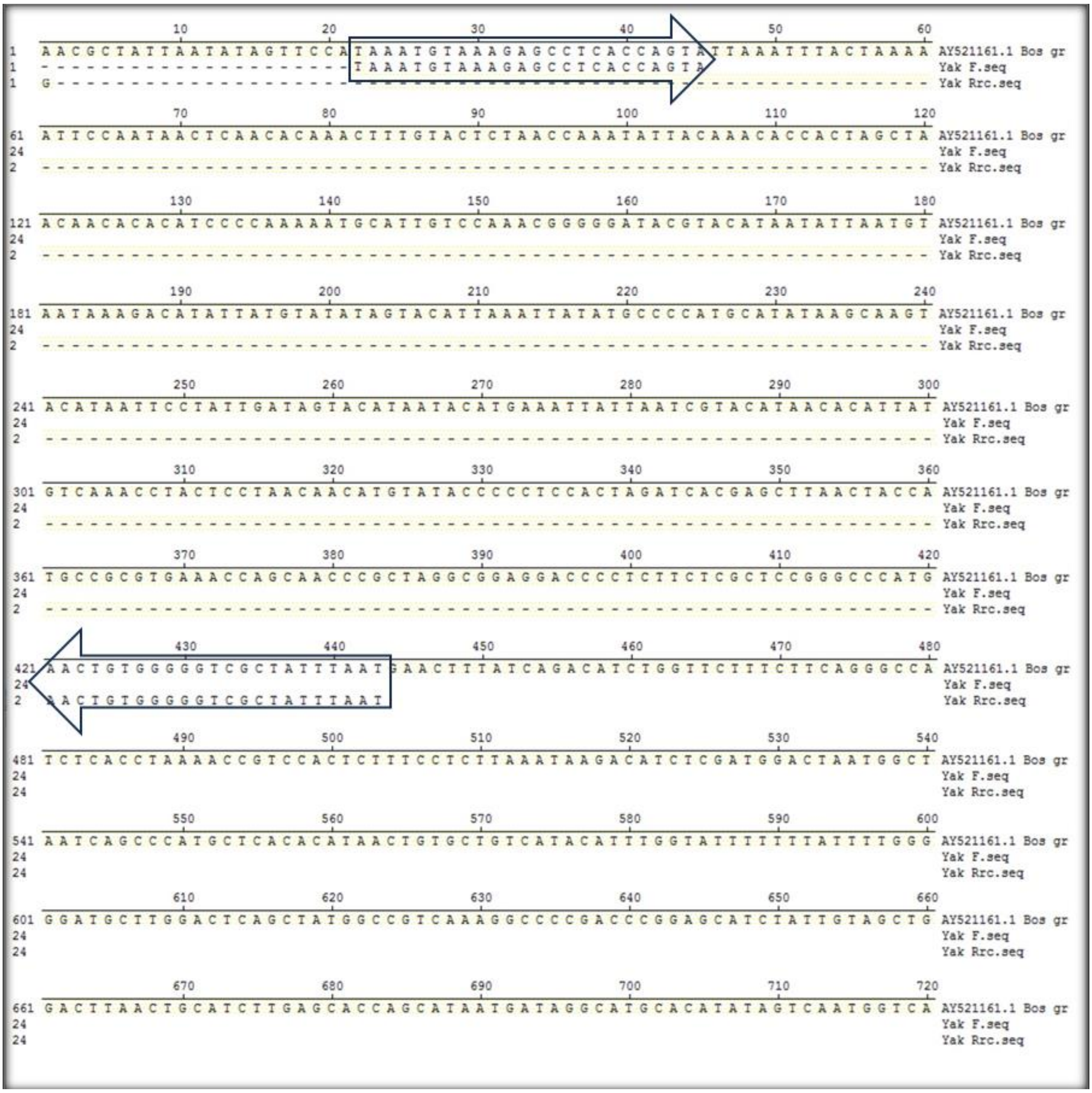

Figure S2. The primer sequences for Yak (*Bos grunniens*)
